# Introductory lecture: Pollen food allergy syndrome

**DOI:** 10.1186/2045-7022-1-S1-S35

**Published:** 2011-08-12

**Authors:** Riccardo Asero

**Affiliations:** 1 Ambulatorio di Allergologia, Clinica San Carlom Paderno Dugnano (MI), Italy

## 

The term pollen-food allergy syndrome (PFAS) defines a series of clinical symptoms appearing shortly after the ingestion of plant-derived foods in subjects with pollen allergy. The patients with PFAS are primarily allergic to pollen and subsequently react to food allergens as a consequence of the homology between pollen and plant-food proteins. The two highly conserved proteins responsible for the large majority of cases of pollen-food allergy syndrome are the pathogenesis-related proteins group 10 (PR-10), including the major birch pollen allergen Bet v 1 and homologous proteins in different fruits and vegetables, and profilin, a plant pan-allergen present in cell structure of all the vegetable kingdom. Although it has been generally thought that the clinical expression of the pollen food-allergy syndrome is uniquely the so-called “oral allergy syndrome”, recent reports suggest that the ingestion of particular foods may be associated with systemic symptoms as well. Recombinant PR-10 proteins and recombinant profilins from different sources are presently available for diagnostic purposes. The presentation will review the available data about the clinical expression, diagnosis, and therapy of the pollen-food allergy syndrome.

